# The mechanisms, epidemiology, and clinical implications of thyroid hormones and age-related macular degeneration: a narrative review

**DOI:** 10.3389/fmed.2026.1765758

**Published:** 2026-01-22

**Authors:** Xiaoling Zhang, Zixun Wang, Zhiqing Li, Zongyue Zhan

**Affiliations:** 1 Handan Eye Hospital (The Third Hospital of Handan), Handan, Hebei, China; 2 Tianjin Key Laboratory of Retinal Functions and Diseases, Tianjin Branch of National Clinical Research Center for Ocular Disease, Eye Institute and School of Optometry, Tianjin Medical University Eye Hospital, Tianjin, China; 3 Shanghai University Affiliated Heping Eye Hospital, Shanghai, China

**Keywords:** age-related macular degeneration, AMD, Mendelian randomization, retinal pigment epithelium, thyroid hormones

## Abstract

Age-related macular degeneration (AMD) is a leading cause of irreversible vision loss among older adults. Thyroid hormones (THs) are essential endocrine regulators of development and metabolic homeostasis, and increasing evidence suggests that TH signaling is involved in retinal physiology and AMD pathogenesis. Experimental studies have demonstrated that excessive TH signaling exacerbates oxidative stress, mitochondrial dysfunction, and apoptosis in retinal pigment epithelium (RPE) cells and photoreceptors, whereas inhibition of TH signaling confers retinal protection in animal models of dry AMD. Genetic evidence from Mendelian randomization analyses further indicates that genetically predicted higher free thyroxine (FT_4_), per one standard deviation increase, is associated with an increased risk of AMD (OR 1.19, 95% CI 1.06–1.33), while no causal association has been established for thyroid-stimulating hormone (TSH). Consistently, large population-based cohort studies, including the Rotterdam Study, have reported a positive association between circulating FT4 levels and the incidence of AMD. In this narrative review, we summarize and critically evaluate recent advances from basic experimental, genetic, and clinical studies on the relationship between thyroid hormones and AMD, discuss potential biological mechanisms underlying this association, and highlight current limitations and future research directions.

## 1 Introduction

Age-related macular degeneration (AMD) is one of the leading causes of irreversible vision impairment and loss among older adults (1). Vision impairment and loss caused by AMD imposes a huge economic burden and severely reduces the quality of life (2). Current cutting-edge research confirms the involvement of multiple factors, such as complement, lipids, angiogenesis, inflammation, extracellular matrix remodeling, and metabolomics, in the pathogenesis of AMD (3–9). Thyroid hormone (TH) is an important endocrine messenger for normal development and physiological function. In the retina, TH signaling plays a critical role in regulating optic protein expression in cone cells. In animal models of AMD, THs have been shown to play an important role in the development of the disease, and anti-TH drugs protect the retinal pigment epithelium (RPE) and photoreceptors from damage and apoptosis (9, 10). Therefore, an in-depth study of the relationship between TH signaling and the pathogenesis of AMD and is of great importance for understanding disease pathogenesis and identifying new therapeutic measures. In this study, we provide a narrative review of the literature, existing shortcomings, and future research directions on the relationship between TH and AMD from three dimensions: basic experiment, genetics, and clinical studies.

This narrative review employed a structured but non-systematic approach to literature identification and synthesis. While not adhering to the complete PRISMA guidelines for systematic reviews, we aimed to minimize selection bias through the following steps: We searched PubMed, Web of Science, and Embase databases for articles published between January 1990 and March 2024 using combinations of the terms “thyroid hormone,” “thyroxine,” “TSH,” “FT4,” “thyroid disease,” and “age-related macular degeneration” or “AMD.” Only studies published in English were considered due to practical constraints in translation and consistency in terminology interpretation. Articles were screened based on relevance to the research theme. Eligible evidence included basic experimental studies, genetic analyses, epidemiological cohorts, case–control studies, and clinical investigations directly examining the relationship between thyroid function, thyroid-related medications, and AMD. The exclusion criteria included non-English publications, conference abstracts without full text, and studies not directly addressing the TH–AMD relationship. Reference lists of relevant reviews and included studies were also screened to identify additional publications.

## 2 Progress in basic research on TH regulation of AMD pathogenesis

### 2.1 Dry AMD, RPE, NaIO_3_, and TH

Ding et al. conducted a series of explorations on the mechanisms by which the TH signaling regulates AMD-related pathological damage. In 2014, the team demonstrated, for the first time, that TH signaling is an important regulator of retinoid expression in optic cone cells (9). Dry AMD is an irreversible degenerative lesion following oxidative damage to photoreceptors and RPE cells. In 2020, the team further demonstrated the effect of TH inhibition on RPE and photoreceptor damage or apoptosis in a mouse model of oxidative stress-induced AMD. Mice treated with sodium iodide (NaIO_3_) exhibited severe RPE and photoreceptor cell death, destruction, and oxidative damage, leading to reduced retinal function. This study demonstrates that the induction of NaIO_3_ by a single injection involves not only oxidative stress and inflammatory response but also the upregulation of apoptotic genes in the RPE and retina. Oxidative stress was the major response in the RPE, whereas apoptosis and inflammation were the major responses in the retina. Inhibition of TH signaling by antithyroid drugs effectively suppressed the expression of these genes in the RPE and retina, thereby reducing oxidative damage in the RPE and photoreceptor cells and maintaining retinal function. The protection of mitochondrial homeostasis *in vivo*, in which the anti-oxidative stress effect is observed, may be central to the protection induced by TH signaling inhibition. Studies of TH regulation in the RPE and photoreceptors have focused particularly on mitochondrial homeostasis or stress. This study demonstrates that inhibition of TH signaling in a mouse model of oxidative stress in AMD protects the RPE and photoreceptors from oxidative damage and cell death while suppressing the upregulation of genes involved in oxidative stress and inflammatory responses. The results of this animal model are consistent with clinical findings, suggesting that high free serum TH levels are associated with an increased risk of AMD and support a role for TH signaling in the pathogenesis of AMD. Since both the RPE and photoreceptors are involved in the pathogenesis of the disease and are protected by antithyroid therapy, inhibition of TH signaling could provide a dual benefit in the treatment of AMD (11). In 2022, building on these findings, the team answered the question of whether antithyroid therapeutic agents protect the RPE and photoreceptors from cellular damage and death and reverse the upregulation of the genes involved in cellular stress responses and cell death. The genes involved in the mouse model of AMD induced using NAIO_3_ were thyroid hormone receptor a1 (*Thra1^−/−^*), thyroid hormone receptor b (*THRB^−/−^*) (which can be expressed in the RPE and inner retinal layers), or thyroid hormone receptor b2 (*THRB2^−/−^*) (which is expressed only in optic cone cells) knockout mice. The results revealed that THR deficiency significantly reduced cell injury, death, upregulation of genes involved in the cellular oxidative stress response, activation of cell death signaling, and the inflammatory response. Deletion of the *Thra1^−/−^*, *THRB^−/−^*, or *THRB2^−/−^* protected against the action of the RPE and photoreceptors, with deletion of the *THRB2^−/−^* genes protecting optic cone cells. In addition, it was shown that *THRB1^−/−^* deletion may have a protective effect on optic rod cells. Importantly, these findings indicate receptor subtype–specific protective effects, with Thra1 deletion predominantly preserving rod photoreceptors, whereas THRB2 deletion conferred protection to both rod and cone receptors. Furthermore, in cell culture, thyroid hormone receptor (THR) antagonists effectively reduced NaIO_3_-induced apoptosis in gene-edited ARPE-19 cells, a spontaneously formed RPE cell line, and human RPE cells. The results of the above studies support the role of THR signaling in the pathogenesis of AMD and the possibility that local inhibition of THR signaling protects the RPE and all other layers of the retina in a dry AMD model (12).

In summary, the above research results showed that the TH signaling pathway is related to the clinical risk of AMD and inhibiting it has a protective effect on AMD lesions; at the same time, different THR subtypes mediated specific protective functions.

### 2.2 Photoreceptor cells, triiodothyronine, and THRB

Recently, the team’s findings provided more direct evidence of the mechanism of triiodothyronine (T_3_)'s damaging effects on the RPE and photoreceptor cells. In this study, *C57BL/6, Thra1^−/−^*, *THRB2^−/−^, THRB^−/−^*, and cone cell dominant *Nrl^−/−^* mice were treated with T_3_ for 30 days and subsequently assessed for retinal function, photoreceptor survival or apoptosis, and retinal damage. Compared with untreated controls, the T_3_-treated group had reduced photoreceptor responses in optic rod and cone cells, reduced outer nuclear layer thickness, and reduced cone cell density. Retinal sections showed a significant increase in cytokines mediating damage and apoptosis. Genetic analyses showed a significant correlation with the upregulation of genes associated with oxidative stress, necrotic-like apoptosis, and inflammation after T_3_ treatment. These results further highlight receptor-specific actions of thyroid hormone signaling, whereby Thra1 predominantly mediates rod photoreceptor vulnerability, while THRB2 plays a broader role in regulating the survival of both rod and cone photoreceptors under excessive T_3_ exposure. The use of antioxidants partially protected photoreceptors and reduced retinal biodynamic tension. Taken together, this study suggests that excessive TH signaling increases necrosis-like apoptosis, exacerbating photoreceptor degeneration and impaired retinal function. The latest study complements this series on the role of TH signaling in retinal degeneration and supports the idea of targeting TH signaling to protect photoreceptors (13).

In addition, the findings of Liu et al. (14) support those of Ding et al., whose results link enhancer activity in the *THRB^−/−^* gene to the sensitivity of optic cone cells to light of different wavelengths. TRβ2 is one of the earliest specific factors induced in the cones. The diversity of cone receptors permits the detection of wavelength information in light, which is the basis of color (chromatic) vision. THs and the THRB gene promote sensitivity to moderately long wavelengths in the retinal organ. Human THR mutations are associated with monochromaticity and impaired response to longer wavelengths. *THRB2^−/−^* also plays a central role in optic cone cell maturation and survival (15). Thus, changes in the *THRB^−/−^* enhancer sequence affecting receptor expression correspondingly alter susceptibility to retinal disease.

**Figure 1:**
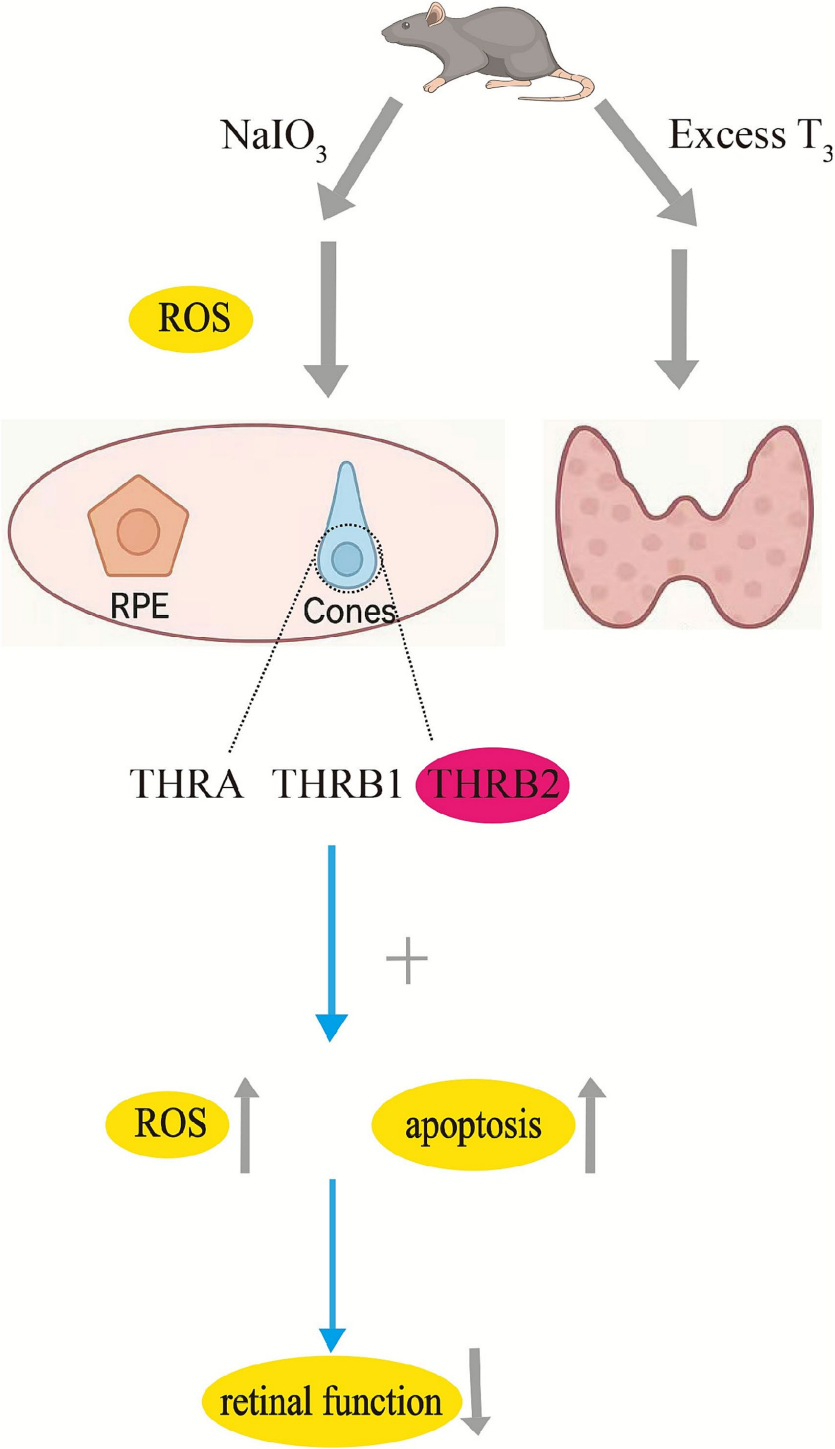
This conceptual diagram illustrates the effects of sodium iodate (NaIO_3_) and excess triiodothyronine (T_3_) on retinal and thyroid-related functions in mice. These induce reactive oxygen species (ROS) production, acting on retinal pigment epithelium (RPE) and cones. Excess T_3_ affects the interaction with thyroid hormone receptors (THR) in the nucleus, in which thyroid hormone receptor a (THRA), thyroid hormone receptor b 1 (THRB1), and thyroid hormone receptor b 2 (THRB2) are involved. The combined action leads to increased ROS and apoptosis, ultimately impairing retinal function. This flowchart was created by the author after organizing the process. This schematic integrates findings from cited studies but remains a hypothesis-generating model for human AMD pathogenesis.

The above results collectively demonstrated that excess T_3_ directly leads to photoreceptor damage and dysfunction, while distinct THR isoforms mediate various damaging effects, with *THRB2* playing a critical role (Figure 1).

## 3 TH regulation and genetic evidence for AMD

Mendelian randomization (MR) is an emerging method of epidemiological research in recent years, both nationally and internationally, to study the exact causal relationship between a disease exposure factor and the disease, with a focus on investigating causal relationships between risk factors and disease outcomes from a genetic perspective (16). Compared with traditional observations and analyses, the use of MR is less susceptible to confounding or reverse causation and supports causal inference in a genetic sense (17–19).

Li et al. (20) included 33,526 individuals (16,144 in the case group and 17,832 controls) from a genome-wide association study (GWAS) of 72,167 Europeans selected for free thyroxine (FT_4_) and TSH-related polymorphisms, with pooled single-nucleotide data on AMD as well as from the GWAS. The results revealed that elevated FT_4_ levels were significantly associated with an increased overall risk of AMD (OR = 1.189, 95% CI = 1.058, 1.334, *p* = 0.005); there was no evidence of a direct causal effect of TSH levels on AMD risk (OR = 0.955, 95% CI = 0.810, 1.125, *p* = 0.582). The above finding suggests that the FT_4_ gene level is positively associated with AMD risk, while there is a lack of clear evidence between TSH and AMD risk. This study establishes, for the first time, a causal relationship between FT_4_ and AMD from a genetic point of view, which is more reliable compared to correlation studies, but this study did not confirm the causal relationship between TSH and AMD. In addition, population studies have revealed that rs943080 in the VEGF-A gene is associated with AMD, and these clinical studies reveal that susceptibility genes may be found in the AMD inflammatory and oxidative stress pathways (21).

## 4 Advances in clinical research on TH regulation and AMD

KG et al. first demonstrated that human RPE cells express TH receptors and speculated that they might be a direct target of TH (22). Recent studies by Tsai et al. (23) have come to a similar conclusion that the two are associated and are emerging as an additional risk factor in the assessment of retinal degenerative diseases (10, 24, 25). Furthermore, a meta-analysis including seven studies (*n* = 61,993) confirmed that thyroid dysfunction was associated with increased AMD risk (pooled OR ≈ 1.19, 95% CI 1.06–1.33; I^2^ = 80.1%, *p* < 0.001), although between-study heterogeneity remained high (26). In contrast, the Blue Mountains Eye Study (*n* ≈ 3,600, 10-year follow-up) did not confirm a consistent association between thyroid function and AMD after multivariable adjustment, particularly for TSH within the reference range (26). Taken together, these findings suggest a stronger and more consistent FT_4_–AMD relationship in the Rotterdam cohort, whereas the Blue Mountains data highlight possible population or methodological differences. The inconsistent associations observed between the Rotterdam Study and the Blue Mountains Eye Study may reflect differences in population characteristics and study design. Variations in dietary iodine intake, age distribution, ethnic composition, baseline thyroid status, and duration of follow-up could influence circulating thyroid hormone levels and retinal vulnerability. In addition, differences in outcome definitions and multivariable adjustment strategies may further contribute to the observed heterogeneity (Table 1).

**Table 1:** Comparative evidence on thyroid function/medication and age-related macular degeneration (AMD).

Study (year, country)	Design	Sample	Exposure definition (TSH/FT4 or medication)	AMD definition	Main effect (OR or HR per study definition, 95% CI)	Adjustments	Risk-of-bias notes	One-line takeaway
Rotterdam Study (2015, Netherlands)	Prospective population-based cohort	Older adults in the Ommoord cohort	Baseline serum FT4 and TSH were measured once and analyzed as continuous variables within the reference range	Incident AMD determined by standardized fundus photography grading (early and late AMD combined)	1.07–1.66	Not specified here	Possible residual confounding; thyroid function assessed only once at baseline; potential regression dilution bias and loss to follow-up	Higher FT4 levels independently predict greater AMD risk of overt thyroid disease.
Blue Mountains Eye Study (2016, Australia)	Prospective cohort (5- and 10-year incidence)	Community adults aged ≥49 years (exact *n* not specified here)	Baseline TSH and FT4 levels were classified into euthyroid and overt thyroid dysfunction categories	Incident AMD over 5- and 10-year follow-up assessed by photographic grading	1.18–3.09	/	Limited number of hyperthyroid cases reducing statistical power; possible misclassification of subclinical thyroid dysfunction; attrition during long-term follow-up	Overt hyperthyroidism increased AMD risk; euthyroid variation did not.
NHIS analysis (2012, USA)	Cross-sectional survey	National Health Interview Survey participants (exact *n* not specified here)	Self-reported physician-diagnosed hypothyroidism (no biochemical confirmation)	Self-reported age-related macular degeneration without ophthalmic confirmation	4.71–17.47	/	High risk of recall and misclassification bias due to self-reported exposure and outcome; cross-sectional design precluding temporality inference	Self-reported hypothyroidism correlated with greater odds of AMD.
Li et al., AJO (2022, multi-country European ancestry)	Two-sample Mendelian randomization	GWAS summary data: thyroid traits (*n* ≈ 72 k) and AMD (*n* ≈ 33 k; exact numbers vary by instrument)	Genetically predicted lifelong FT4 and TSH levels derived from GWAS instruments	Overall, AMD is defined by the GWAS case–control status	FT4: 1.21 TSH: 0.81	Instrumental-variable assumptions; sensitivity analyses for pleiotropy	Potential horizontal pleiotropy despite sensitivity analyses; restriction to European ancestry limiting generalizability	Genetic evidence supports a causal link between higher FT4 levels and increased AMD risk.
Xu et al., Ophthalmic Research (2021, meta-analysis)	Meta-analysis of epidemiologic studies	Seven studies; ~61,993 participants (per manuscript)	Thyroid hormone replacement therapy ascertained by medication records (proxy for treated thyroid disease)	AMD per each included study	0.92–1.72	Study-level adjusted estimates (varied)	Between-study heterogeneity; varying exposure/outcome definitions	Across studies, thyroid disease is associated with increased AMD risk.
Beaver Dam Eye Study (2013, USA)	Prospective cohort (5-year incidence of early ARM)	Community cohort (exact *n* not specified here)	Medication use, including thyroxine	Early age-related maculopathy (ARM), not equivalent to late-stage AMD	1.10–1.47	/	Substantial confounding by indication, as thyroid hormone use reflects underlying thyroid disease severity rather than hormone exposure alone; outcome limited to early ARM rather than late AMD	Medication-based evidence is inconclusive due to confounding by indication.
KORA-Age Study (2017, Germany)	Population-based cross-sectional/registry analysis	Older adults in the Augsburg region	Thyroid hormone treatment	Common eye diseases (AMD not isolated)	1.11–4.53	/	Substantial confounding by indication, as thyroid hormone treatment reflects underlying thyroid disease severity rather than hormone exposure alone; non-specific ocular outcomes; cross-sectional design	Signals for ocular risk with TH therapy, but AMD-specific inference is limited.

OR, odds ratio; HR, hazard ratio; CI, confidence interval. Effect estimates are reported as originally presented in each study and are not directly comparable across study designs.

Both clinical and subclinical thyroid dysfunction are common in the general population. In Europe, the collective prevalence of these conditions reaches a level of 3.82%. However, the mechanisms linking thyroid disorders to AMD are unclear. TH are potent regulators of mitochondrial biogenesis and oxidative phosphorylation. Elevated TH signaling can increase mitochondrial respiration and ATP production, thereby enhancing ROS generation. In RPE cells, which exhibit high metabolic demand and limited antioxidant capacity, this shift may overwhelm protective enzymes such as superoxide dismutase and glutathione peroxidase, resulting in oxidative damage and cellular dysfunction. This mechanism provides a plausible biological link between elevated TH activity and increased susceptibility to AMD.

Retinal tissue exhibits susceptibility to oxidative stress, with such damage being a well-established constituent of AMD’s multifaceted pathogenesis. Furthermore, population studies indicate that certain VEGF-A genes are linked to AMD and hold relevance in association studies focusing on TSH ranges. A possible explanation may be the discovery of sensitivity genes in inflammatory and oxidative stress pathways. Additional studies have confirmed that thyroid dysfunction is associated with vascular function, lipids, and atherosclerosis (27) and is a susceptibility factor for the development of AMD (28).

In addition, several clinical studies have explored the perspective of thyroid medications and the occurrence of AMD (10, 25). Thyroxine medications also appear to play a role in influencing the risk of developing AMD, with a population-based study in the Augsburg region reporting a significantly higher risk of other ocular disorders in TH-treated male patients, but the relationship is unclear at this time and needs to be validated by numerous later prospective studies and large samples of data. A separate meta-analysis of 118,420 participants examining thyroid hormone replacement therapy also indicated an association with AMD, but with moderate heterogeneity (I^2^ = 69.0%) (25). In contrast, a study of a large population-based sample of adults aged 50 years showed a correlation between hypothyroidism and AMD, particularly among white women (29). Future studies with larger sample sizes are needed.

## 5 Treatment and summary

Recent therapeutic advances have highlighted the role of nuclear receptors in regulating aging-related pathways, including oxidative stress, inflammation, lipid metabolism, and angiogenesis, many of which are implicated in AMD pathogenesis (30). However, accumulating experimental evidence indicates that excessive or systemic TH signaling exacerbates oxidative damage and mitochondrial dysfunction in RPE and photoreceptors, suggesting that global activation of TH pathways is unlikely to be beneficial (30).

Importantly, the therapeutic relevance of TH receptors should not be interpreted as supporting systemic receptor agonism. Instead, future strategies may involve selective, tissue-specific, or context-dependent modulation of nuclear receptor signaling, aiming to preserve physiological homeostatic functions while avoiding the detrimental effects associated with excessive TH activity (30). At present, there is insufficient evidence to support TH receptor agonists as a therapeutic option for AMD, and local inhibition or fine-tuned regulation of TH signaling appears more consistent with existing experimental findings.

Furthermore, AMD is a complex, multicellular disease involving not only RPE cells but also photoreceptors, choroidal endothelial cells, resident immune cells, and microglia. A comprehensive understanding of nuclear receptor signaling across these cellular compartments will be essential before any receptor-based interventions can be translated into clinical practice (23).

In conclusion, this study describes the correlation between TH and AMD in recent years and new ideas of treatment mechanisms from the perspectives of basic experiments, clinical research, genetic method research, etc. However, due to the complexity of the mechanism of AMD disease itself, coupled with the fact that the current relevant research on the mechanism of hormone-induced AMD doctrine is still in the stage of discovery of the correlation, residual confounding factors cannot be excluded, and the underlying mechanisms of this association remain speculative—whether through direct retinal TH signaling, systemic oxidative stress, vascular alterations, or shared genetic susceptibility, it is not yet clear. Consequently, the lack of TH axis-mediated ocular AMD occurs in the stage of research or remains in the stage of extrapolation. It is believed that, in the future, with the continuous improvement of exploring the specific mechanisms of the two, breakthroughs will be made in the precise treatment of dry AMD.

Some studies have adopted telomere length (TL) and age-related phenotypes as indicators of senescence. As a biomarker of cellular aging, TL is also recognized as a risk factor for age-related diseases. Given that telomere length shortens with advancing age, it has been extensively explored in relation to both cellular senescence and the risk of developing age-related diseases.

The current review includes studies that inevitably have several limitations: First, unmeasured factors such as diet, systemic inflammation, or comorbidities may influence thyroid status and AMD risk despite multivariate adjustment. Second, a majority of studies did not distinguish between early and late AMD or between dry and neovascular AMD, which may have weakened the association. Third, a majority of cohorts assessed FT_4_/TSH once at baseline, which may not capture lifetime thyroid function or fluctuations. Fourth, a majority of genetic and cohort data are from European populations; there is limited and sometimes discrepant evidence for Asian and other ancestries. In addition, the observed associations with thyroid medications may reflect, in part, the severity or comorbidity of the underlying thyroid disease rather than the drug effect itself. Finally, formal quality assessment tools (e.g., Newcastle–Ottawa Scale, ROBINS-I) were not applied. Future studies should prioritize multi-ethnic MR analyses and large prospective cohorts in non-European populations, particularly in Asian and iodine-variable regions, to evaluate the generalizability of the observed associations and to explore potential ancestry-specific or environmental modifiers of thyroid hormone–AMD relationships.

Studies have shown that thyroid disease accelerates the onset of age-related phenotypes, including shortened TL, premature onset of senile cataracts, and AMD, and further experimental studies could be conducted in the future to elucidate the mechanisms by which thyroid disease promotes aging, which could help to slow the progression of aging and disease (31). It is also worth noting that internists and endocrinologists often miss ophthalmological treatment before referring patients with thyroid disease to ophthalmologists, and this delay in referral may potentially lead to prolonged treatment of thyroid eye disease. It is inferred that its optimal treatment may also be missed in the case of AMD, which needs to be studied in the future (32). Based on previous studies, we found that genetically predicted higher FT_4_ (within the normal range) was associated with later AMD, and we found that this association was predominantly evident in women. However, these may be serendipitous findings, and high normal FT_4_ levels may increase the risk of AMD later in life, especially in women, and further research is needed to investigate the role of FT_4_ in AMD and potential gender differences (33).
